# Linking Hydrogen (*δ* ^2^H) Isotopes in Feathers and Precipitation: Sources of Variance and Consequences for Assignment to Isoscapes

**DOI:** 10.1371/journal.pone.0035137

**Published:** 2012-04-11

**Authors:** Keith A. Hobson, Steven L. Van Wilgenburg, Leonard I. Wassenaar, Keith Larson

**Affiliations:** 1 Environment Canada, Saskatoon, Saskatchewan, Canada; 2 Department of Zoology, Lund University, Lund, Scania, Sweden; University of Regina, Canada

## Abstract

**Background:**

Tracking small migrant organisms worldwide has been hampered by technological and recovery limitations and sampling bias inherent in exogenous markers. Naturally occurring stable isotopes of H (δ^2^H) in feathers provide an alternative intrinsic marker of animal origin due to the predictable spatial linkage to underlying hydrologically driven flow of H isotopes into foodwebs. This approach can assess the likelihood that a migrant animal originated from a given location(s) within a continent but requires a robust algorithm linking H isotopes in tissues of interest to an appropriate hydrological isotopic spatio-temporal pattern, such as weighted-annual rainfall. However, a number of factors contribute to or alter expected isotopic patterns in animals. We present results of an extensive investigation into taxonomic and environmental factors influencing feather *δ* ^2^H patterns across North America.

**Principal Findings:**

Stable isotope data were measured from 544 feathers from 40 species and 140 known locations. For *δ* ^2^H, the most parsimonious model explaining 83% of the isotopic variance was found with amount-weighted growing-season precipitation *δ* ^2^H, foraging substrate and migratory strategy.

**Conclusions/Significance:**

This extensive H isotopic analysis of known-origin feathers of songbirds in North America and elsewhere reconfirmed the strong coupling between tissue *δ* ^2^H and global hydrologic *δ* ^2^H patterns, and accounting for variance associated with foraging substrate and migratory strategy, can be used in conservation and research for the purpose of assigning birds and other species to their approximate origin.

## Introduction

The ability to track organisms through time and space by various means has revolutionized our understanding of key evolutionary processes shaping the life histories of migratory organisms [1]. Understanding the geographical connectivity among various stages of the annual cycle of migrants has tremendous benefits towards implementing effective conservation strategies [2]. Advances in migrant research have largely been due to technological devices, such as light-sensitive geolocators affixed to animals [3], [4], and breakthroughs are expected through the use of satellites that can detect and track small VHF transmitters on animals (http://icarusinitiative.org/index.html).

Despite exciting technological advances in the use of attached markers to track ever more and smaller organisms remotely or through recapture, external methods unfortunately remain biased to location of effort and small sample sizes. Conversely, intrinsic, naturally occurring molecular or stable isotope markers of origin are comparatively inexpensive, and are unbiased by the restricted distribution of tagged individuals, hence large samples can be obtained in which every individual can be used to infer origins [5]–[7].

Among isotopic markers, stable-hydrogen isotopes in animal tissues, especially feathers of birds (*δ* ^2^H_f_), has proven particularly successful because well-known and predictable continental patterns of hydrogen isotopes in rainfall (*δ* ^2^H_p_) are often closely reflected in tissue *δ* ^2^H values. Isotopic locational information is stored in tissues, particularly those that are metabolically inert following growth (e.g. feathers, hair, claws; [8]). Simply put, tissue *δ* ^2^H values can be compared to hydrogen isotopic patterns (isoscapes) in order to infer origins where tissues were grown. For birds, the assignment of individuals and populations to their region of origin using feather *δ* ^2^H_f_ isoscapes has proven to be a leading advance in establishing migratory connections, especially for migrant and threatened species in North America [9]–[13] and Europe [14]–[16].

Since the first studies demonstrating the potential of the *δ* ^2^H_f_ assignment approach [8], [17], advances have been made using GIS and Bayesian statistical techniques to derive probabilistic isotopic regions of animal origins [13], [16], [18], [19]. These new approaches propagate all known associated errors from the isotopic analyses to deviations from the *δ* ^2^H_p_ isoscapes on which they are based [20], and thereby provide a more defensible assessment of probable origin, compared to previous regression-based origin mapping. Increasingly, spatial isotopic data from known origin birds and animals are being used to improve and inform the primary algorithms in probabilistic geographic assignment models (reviewed in [6], [21]). Despite ongoing advances, a few authors have doubted the accuracy of the intrinsic marker approach and specifically H isotopes, pointing to potential sources of error that are either not well understood or were poorly handled in the early assignment methods [22]–[25]. A significant concern was that fundamental precipitation-based hydrologic patterns of deuterium (^2^H), that underpin H isotopic flow into food webs, is based on long-term averages obtained from a 50-year disparate global record, and that it remains unclear how much interannual differences at any location vary from this long term. Secondly, with ongoing research it became clear that no single global assignment algorithm is appropriate for all species, taxa, or age classes [6] as a number of ecophysiological factors may play a role in H isotopic discrimination ultimately linking tissue and rainfall [26], and that species-specific H-isotope tissue base maps may be required. These apparent H isotope difference among animals are ultimately linked to possibly numerous processes affecting the H isotope budget of individuals, most of which remain poorly understood [27]. For example, adult birds that overlap molt with breeding can experience higher metabolic rates and water loss, and thus may result in higher *δ* ^2^H_f_ values than their sedentary young [28]. In addition, ecologically dissimilar species may sample foodwebs controlled by different components of the hydrological cycle even in the same general area (e.g. wetlands vs. uplands) and so show different *δ* ^2^H_f_ values. Further, for some species like carnivores the H isotopes in their tissues have been shown to be almost completely disconnected from the underpinning spatial H isotope patterns [29]. While most of these challenges can be overcome by constructing species-specific or ecologically equivalent natal H isotope tissue basemaps within the timeframe of a study [21], this requires a herculean effort for most species, and access to spatially extensive and remote areas for many Neotropical migrants remains a huge challenge. Thus, efforts to obtain assignments for groups of “like” organisms remains an ongoing area of research.

Hobson and Wassenaar [8] derived the first H isotope assignment algorithm for insectivorous passerines in North America by sampling adult breeding birds across a latitudinal gradient. They assumed those individuals grew their flight feathers at those sampled locations the year before, but were unable to control for possible natal dispersal into those populations. Other studies, such as [30] established a H isotope assignment base map or isoscape for several species of raptors in North America. They based their sampling on juvenile birds that were closely associated with sampling locations, but it later became clear that such an isoscape cannot necessarily be used for carnivorous adult raptors [28]. Since these attempts, basemap isoscapes have been constructed for waterfowl [31] and European species [26], but it is still uncertain how generalizeable these feather isoscapes are. This uncertainty remains the single greatest impediment to the broader application of H isotope techniques to estimate of origins of migratory birds.

Here, we obtained and measured H isotopes in a large collection of feathers from across North America, which included a new group of songbird individuals that were known with certainty to have grown their feathers at specific sites across contiguous North America. Our objective was to construct the best possible calibrated feather isoscape for passerines and other species in North America and to further quantify what biotic and abiotic factors may influence isotopic patterns across large geographic scales.

## Methods

### Ethics statement

This study was conducted under authority of the Canadian Council for Animal Care as reviewed by the University of Saskatchewan (Permit 20100084). All federal permits for the use of feather material were issued by the Canadian Wildlife Service and the U.S. Fish and Wildlife Service.

### Samples

**Figure 1:**
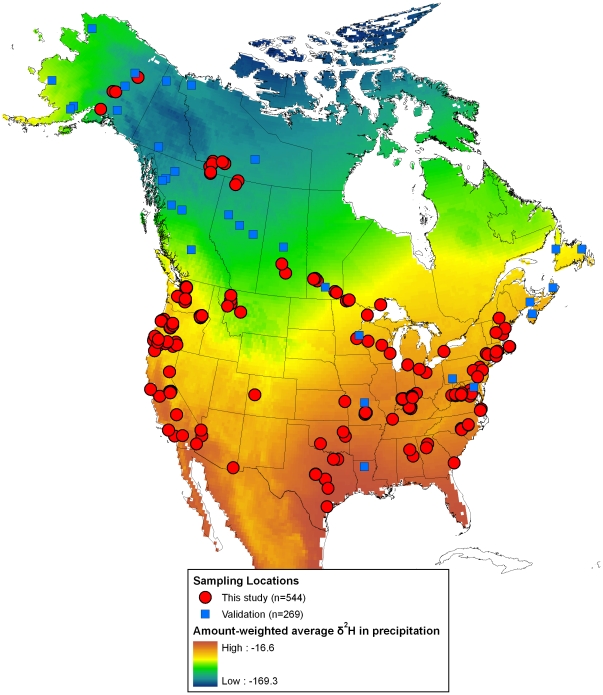
Location of feather sample collection sites and the underlying gradient in amount-weighted average *δ* ^2^H in precipitation. Shown are locations for 544 calibration samples collected for this study, and the location of 269 samples from previously published sources used as model validation samples. Underlying isoscape based on Bowen et al. [20].

The majority (n = 434 of 544) of feathers sampled were outer-tail feathers (rectrices) of passerines collected during the breeding season at constant-effort mist-netting sites such as MAPS (Monitoring Avian Productivity and Survivorship, [32]) and by other groups across North America. These samples were selected from a collection stored at the Center for Tropical Research (CTR) at University of California Los Angeles (UCLA). Feather samples for analysis were selected from two databases at CTR, one maintained for the study of migratory connectivity related to studies of avian influenza (N = 56443; database version: 2009.10.12) and another called “Neotrops” (N = 57468; database version 2009.06.18) used for the study of migratory connectivity generally. We queried these databases to select only individuals that were captured during the breeding season (May to July) and in at least two different years at the same location. This “recapture” criterion was used to identify individuals that breed at a single location. We further restricted feather sample selection to birds in which the prior capture occurred in the year immediately prior to the sample collection, hereby ensuring that we could be certain of the exact location of feather growth. Birds that were captured during their hatching year and then recaptured in subsequent years were eliminated to avoid confounding age effects. The locations of these sampling sites are shown in Fig. 1.

In addition, sampling was restricted to species known to have a complete pre-basic (post-breeding) molt on the breeding grounds [33], namely the annual molt of their flight feathers occurring on the breeding grounds, to avoid potential complication of inadvertently sampling a feather grown the previous years. This careful screening results in some spatial data gaps, thus 60 samples were included from species that are known to occasionally undergo molt-migration or molting during migration and hence away from the breeding grounds; however, data from these samples were carefully examined for outliers. The locations of these sample sites are shown in Figure 1.

In addition to known origin samples obtained from the CTR sample collection, first primary (P1) samples were obtained from 91 samples collected at 16 sites in Canada. These included 25 samples from after-second year Ovenbirds (*Seiurus auricapilla*) for which it was assumed they held the same territory between years. Samples were obtained from 33 recaptured Golden-winged Warblers (*Vermivora chrysoptera*) from a mark-recapture experiment, and 30 hatching-year Tree Swallows (*Tachycineta bicolor*) captured near their nesting location. The locations of these sample sites are included in Figure 1 and a description of the species used are provided in Table S1.

### Stable isotope analyses

All feathers were cleaned of surface oils in 2∶1 chloroform∶methanol solvent rinse and prepared for *δ* ^2^H analysis at the Stable Isotope Laboratory of Environment Canada, Saskatoon, Canada. The impact of exchangeable hydrogen was corrected by conducting hydrogen isotope analyses using the comparative equilibration method described by [34] and using three calibrated keratin hydrogen-isotope reference materials. H Isotopic measurements were performed on H_2_ gas derived from high-temperature (1350°C) flash pyrolysis of 350±10 ug feather subsamples from the distal section of feather vane and keratin standards using continuous-flow isotope-ratio mass spectrometry. Measurement of the three keratin laboratory reference materials (CFS, CHS, BWB) corrected for linear instrumental drift were both accurate and precise with typical mean *δ* ^2^H ± SD values of −147.4±0.79‰ (*n* = 5), −187±0.56‰ (*n* = 5) and −108±0.33‰ (*n* = 5), respectively. A control keratin reference yielded a 6-month running SD of ±3.3‰ (*n* = 76). All results are reported for non-exchangeable H expressed in the typical delta notation, in units of per mil (‰), and normalized on the Vienna Standard Mean Ocean Water – Standard Light Antarctic Precipitation (VSMOW-SLAP) standard scale.

### Modeling variation in *δ* ^2^H_f_

Numerous studies have demonstrated the causal link between *δ* ^2^H in rainfall and animal tissues, however, we wished to determine if various life history traits influence this relationship. Thus, life history data was obtained from the Avian Life History Information Database (http://www.on.ec.gc.ca/wildlife/wildspace/project.cfm), or from species accounts in the Birds of North America [35]. Using these data, the species were categorized into 1) foraging guild (insectivore, omnivore), 2) foraging substrate (ground foragers versus species foraging elsewhere (in shrubs, canopy or aerially; hereafter non-ground foragers)), 3) migratory guild (Neotropical migrant, short-distance migrant, resident), and 4) whether the species was associated with an aquatic versus upland habitat. These life history data were used as factors in all subsequent statistical modeling. As we discuss in more detail below, while not exhaustive, our choice of parameters for modeling variation in *δ* ^2^H_f_ were based on our expectation that trophic position could influence *δ* ^2^H_f_, that foodwebs associated with the soil-leaf litter interface may be enriched in ^2^H over the canopy and shrub layers and that migratory strategy is associated with timing of molt and hence period of isotopic integration. Finally, at continental scales, aquatic environments may represent different hydrogen pools available to birds compared to averaged precipitation.

Prior to modeling spatial variation in *δ* ^2^H of feathers, alternative models of *δ* ^2^H in the underpinning precipitation isoscapes were examined. Previous work on North American migratory birds largely focused on precipitation amount-weighted growing season average *δ* ^2^H in precipitation (namely, based on months where the mean daily temperature was >0°C; hereafter *δ* ^2^H_p_), and this was included as one estimate of local *δ* ^2^H in precipitation. However, it remains unclear which months of rainfall exert the greatest influence driving H isotopic flow into primary foodwebs that contribute to avian diet and feather growth. Thus, the relationship between *δ* ^2^H_f_ and amount-weighted average *δ* ^2^H in precipitation for the months of May through July (hereafter *δ* ^2^H_pMay–July_) time period were also examined. Model-based estimates of *δ* ^2^H_p_ were obtained from the geospatial model provided by [20]. Estimates of *δ* ^2^H_pMay–July_ were obtained from a geospatial model of *δ* ^2^H in May–July precipitation using IsoMAP [36], using CRU-derived climatic variables [37] to model *δ* ^2^H in precipitation. During the preliminary analysis it was observed that *δ* ^2^H_f_ was more strongly correlated with *δ* ^2^H_p_ (Pearson r = 0.88, p<0.001) than *δ* ^2^H_pMay–July_ (r = 0.73, p<0.001) thus all subsequent modeling was done using *δ* ^2^H_p_ as a linear covariate and *δ* ^2^H_pMay–July_ was not considered further.

General Linear Models (GLM) were used to statistically model variation in *δ* ^2^H_f_ from a calibration set of 544 known origin birds from across North America (Figure 1). A preliminary model found only two individual outliers, which were removed prior to subsequent modeling. Of the two outliers, one was significantly depleted (−81.7‰) relative to isoscape predictions, while the other was substantially more enriched (58.4‰) than expected. Examination of the data suggest that the depleted sample likely derived from a food web altered by irrigation from the Colorado River (Williamson County, Texas), whereas the enriched sample was from a riparian associated species (MacGillivray's warbler, *Oporornis tolmei*) sampled near the Umatilla River in Oregon.

Twenty five *a priori* candidate GLM models were considered, and the most parsimonious model was selected based on AIC*_c_* (Akaike's Information Criterion adjusted for small sample size) and on AIC model weights [38]. With the exception of an intercept only model, the candidate models included *δ* ^2^H_p_ as the predictor variable; but varied with the inclusion of those factors for foraging guild, foraging substrate, migratory guild, and age class (hatching-year and second-year birds versus after hatching-year) and interactions up to the second order.

### Examination of residual variation

Residuals from our top model for variation in *δ* ^2^H_f_ were explored using descriptive statistics and testing for spatial autocorrelation using Moran's index of autocorrelation. Furthermore, residuals from our top model were examined for variation related to body size (wing-length (mm), body mass (grams)), year of feather growth, and climatic departures from long-term averages during the year of feather growth (North Atlantic Oscillation Index (NAO) and multivariate El Niño-Southern Oscillation Index (ENSO)).

### Model validation

We examined how well our top model performed on temporally distinct data from birds of presumed origin from previously published sources [8], [31], [39]. First, 269 observed *δ* ^2^H_f_ values were regressed against predicted *δ* ^2^H_f_ from the top model. In addition, using these same 269 samples, likelihood-based assignment algorithms [40], [41] were used to assign individuals to origins. Briefly, the likelihood-based assignments were conducted by using normal probability density functions to assess the likelihood that an observed *δ* ^2^H_f_ value could have been generated at a given location given the isoscape predicted value for that location and the level of error inherent in the assignment process. The likelihoods were converted to likely (1) versus unlikely (0) origins by selecting areas associated with a threshold probability density value, based on a user specified odds ratio of being correct versus incorrect [13], [41], [42]. We selected odds of 2∶1 and 3∶1 odds respectively (to obtain upper 67 and 75% of cumulative probabilities respectively), and used the standard deviation of our calibration equation residuals as an estimate of error in the likelihood assessment [13], [41], [42]. The frequency at which individuals were correctly assigned to the region from where they were captured were examined. The observed frequencies of correct versus incorrect classifications were contrasted with the expected frequencies that should occur under the selected odds ratios (i.e. 67% and 75% correct for 2∶1 and 3∶1 odds respectively), using a Chi-squared goodness-of-fit test.

## Results

### Variation in *δ* ^2^H_f_

**Figure 2:**
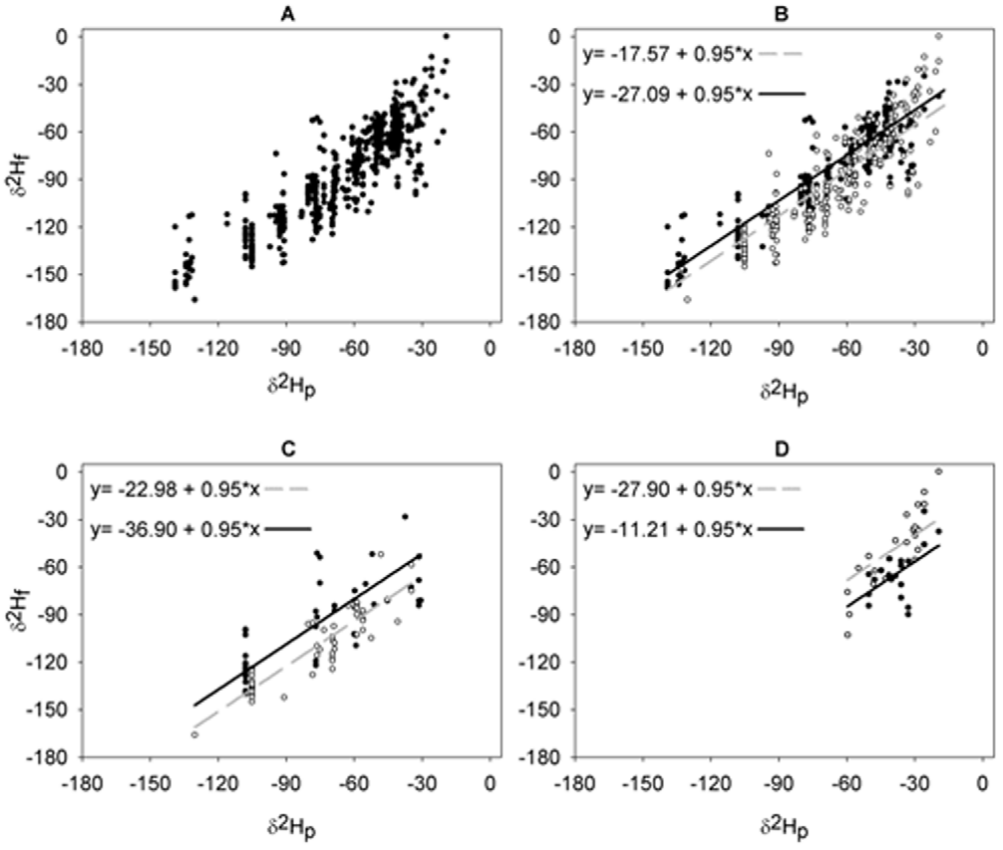
Relationship between *δ* ^2^H_f_ and isoscape predicted *δ* ^2^H_p_. A) For 542 model calibration samples, B) for Neotropical migrants, C) for short-distance migrants, and D) for resident species. The relationship between *δ* ^2^H_f_ and *δ* ^2^H_p_ was modeled using a linear modeling approach with lines indicating predictions from our best AIC**_c_** selected model (see methods). Our best model included a factor for whether the species foraged on the ground or elsewhere; thus, the predictive equations and regression line are shown separately for ground foragers (solid lines) and non-ground foragers (dashed lines).

A strong relationship was observed between *δ* ^2^H_f_ and *δ* ^2^H_p_ (Figure 2A). Of 25 candidate models explored to explain variation in *δ* ^2^H_f_, one model received overwhelming support (Table S2). The most parsimonious model received 99% of the support based upon model weights, and was separated from the next model by over 10 AIC_c_ units. The top model included *δ* ^2^H_p_, migratory guild, foraging substrate and the interaction between migratory guild and foraging substrate as parameters. The resulting model explained a substantial proportion of the variance in *δ* ^2^H_f_ (F_6,535_ = 436, p<0.001, r^2^ = 0.83).

Parameter estimates (Table 1) from the top model were used to derive six predictive equations for estimating *δ* ^2^H_f_ for birds from a given migratory guild and foraging substrate throughout North America (Figure 2 B–D). Each equation had a common slope of 0.95 (0.02 SE) with *δ* ^2^H_p_, but different intercepts based upon parameters for migratory guild, foraging substrate and their interaction (Table 1).

**Table 1:** Parameter estimates for the top linear model examining variation in *δ* ^2^H_f_.

Parameter	Estimate	SE	t	p
Intercept	−17.57	1.65	−10.63	0.00
*δ* ^2^H_p_	0.95	0.02	44.10	0.00
Resident dummy^a^	−10.33	3.15	−3.27	0.00
Short-distance Migrant dummy^b^	−5.41	2.18	−2.48	0.01
Foraging Substrate dummy^c^	−9.52	1.32	−7.20	0.00
Resident*Foraging substrate interaction	26.21	4.31	6.08	0.00
Short-distance Migrant*Foraging substrate interaction	−4.40	2.85	−1.55	0.12

a 1 = Resident, 0 = Neotropical or Short-distance Migrant.

b 1 = Short-distance Migrant, 0 = Neotropical Migrant or Resident.

c 1 = Species foraging in Canopy, shrubs or aerially, 0 = Ground foraging species.

Combining parameter estimates for migratory guild, foraging substrate and their interaction (Table 1) showed that non-ground foraging, short-distance migrants had more negative *δ* ^2^H_f_ values (∼−36.9‰), followed by ground foraging resident species (∼−27.9‰), non-ground foraging Neotropical migrants (−27.1‰), ground foraging short-distance migrants (−23.0‰), ground foraging Neotropical migrants (−17.6‰), and finally by non-ground foraging resident species (−11.2‰; see Figures 2 B–D). Thus, with the exception of resident species, migrant ground foragers generally had higher *δ* ^2^H values than migrant species that do not forage on the ground, with an average hydrogen isotopic enrichment of approximately 9.5‰ for Neotropical migrants (Figure 2B), and approximately 13.9‰ for short-distance migrants (Figure 2C). The opposite pattern was found for resident species, where it was found that ground foragers were more negative by approximately 16.7‰ relative to non-ground foragers.

### Model validation

**Figure 3:**
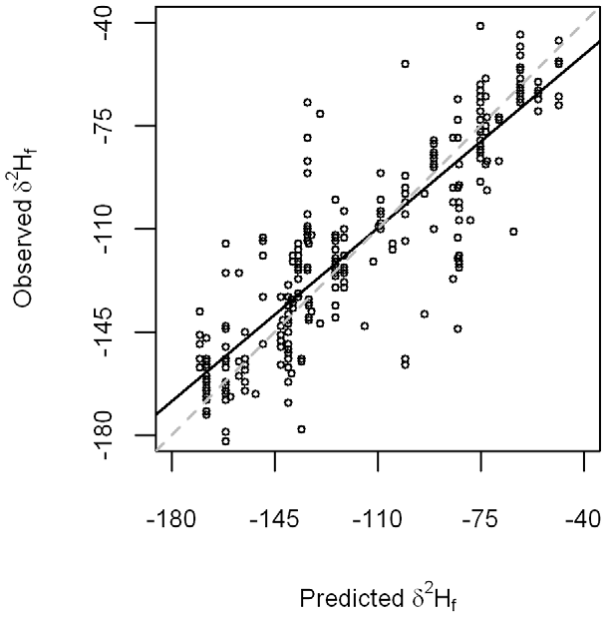
Regression of observed versus model predicted *δ* ^2^H_f_ for 269 samples from previously published sources. Solid line depicts ordinary least squares fit (observed = −17.38 (3.56 SE)+0.84*predicted (0.03 SE)), and dashed line is 1∶1 correspondence line.

We applied our top model (Table 1) to predict *δ* ^2^H_f_ for our external validation data set (n = 269). Regression of observed versus predicted *δ* ^2^H_f_ suggested that our model performed reasonably well (F_1,267_ = 835, p<0.001, r^2^ = 0.76) at predicting *δ* ^2^H_f_ for the validation data set (Figure 3). However, the parameter estimates from this equation suggested some minor bias, with an intercept of −17.4‰ (3.56‰ SE), and slope of 0.84 (0.03 SE).

Applying likelihood-based assignments to determine origin for the 269 validation samples resulted in classification accuracies similar to those expected given the specified level of risk. Selecting GIS cells within the isoscape that corresponded to 2∶1 odds of correct classification resulted in 172 samples being correctly classified to place of capture, and 97 samples assigned to origins that did not include the place of actual capture. By contrast, the expected frequency of correct to incorrect classifications was 179 to 90 samples, which did not differ from the observed frequencies (χ^2^ = 0.40, df = 1, p = 0.53). Similarly, at 3∶1 odds, the observed frequencies of correct (192) to incorrect (77) classifications did not differ from the expected frequencies of 202 and 67 (χ^2^ = 0.95, df = 1, p = 0.33).

### Residual variation

**Figure 4:**
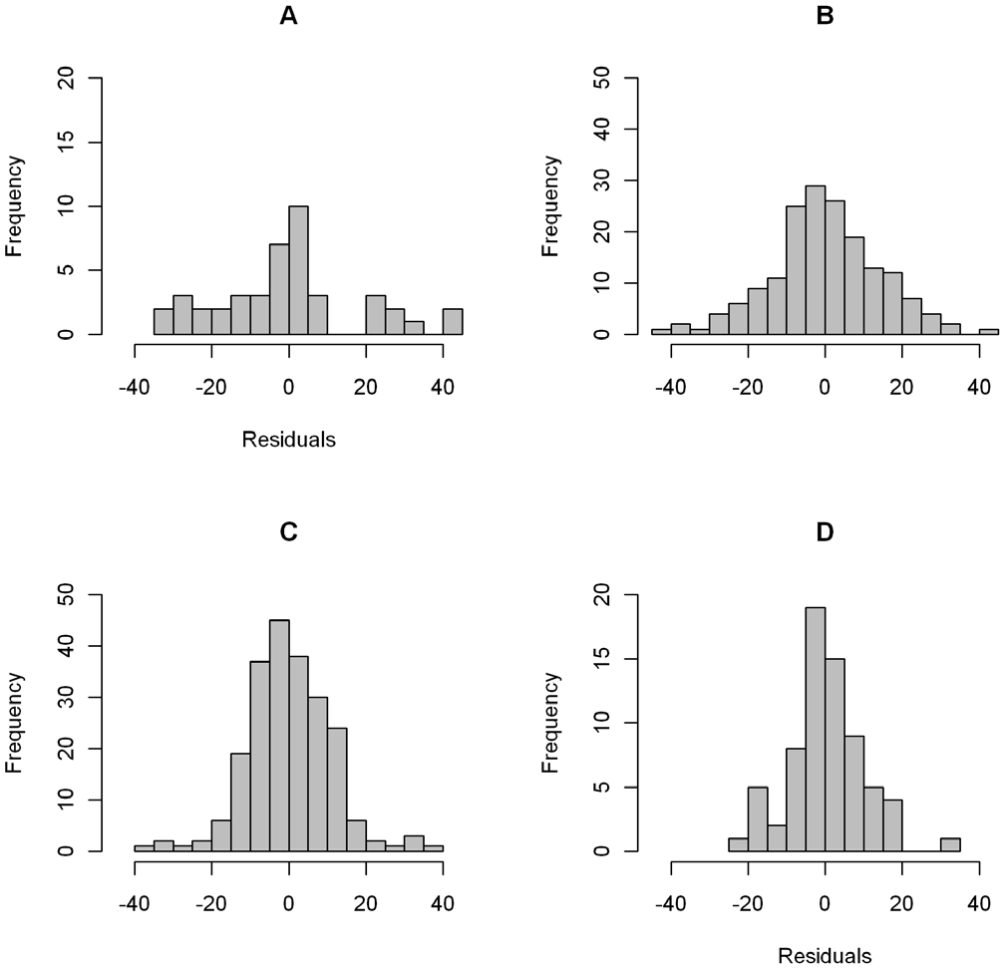
Frequency distribution of residuals from top model. For A) ground foraging short-distance migrants, B) non-ground foraging Neotropical migrants, C) ground foraging Neotropical migrants, and D) non-ground foraging short-distance migrants. Residuals are not shown for resident species.

Residuals for the top model were well distributed (Figure 4). The overall model residuals had a standard deviation of 12.9‰; however, this varied between migratory guild and foraging substrate (Figure 4). The residuals showed the greatest dispersion was for ground foraging short-distance migrants (SD = 18.4‰, Figure 4A), followed by non-ground foraging Neotropical migrants (SD = 14.4‰, Figure 4B), ground-foraging Neotropical migrants (SD = 10.8‰, Figure 4C), and non-ground foraging short-distance migrants (SD = 9.7‰, Figure 4D). Model residuals showed no evidence of remaining spatial variation (Moran's I index = 0.03, z = 0.34, p = 0.37).

**Figure 5:**
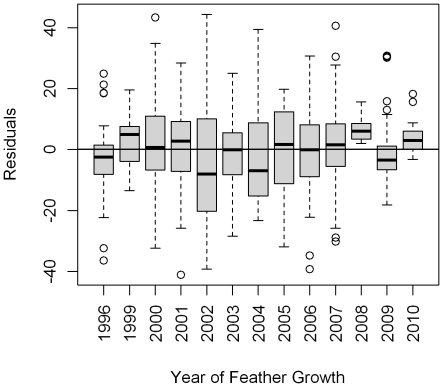
Boxplot of residuals from the top model for *δ* ^2^H_f_ versus the year in which the feather was grown. Dark solid line represents the median, gray box indicates the range inter-quartile range, whiskers are 1.5 time the inter-quartile range, and dots indicate extreme values. Note: only years with at least ten samples are shown.

In general, the model residuals fit the long-term predictions well, displaying relatively minor inter-annual variation relative to the long-term predictions (Figure 5). Graphical examination of the model residuals against wing length, body mass, and the North Atlantic Oscillation index showed no patterns. By contrast, graphical examination of the residuals against ENSO suggested a potential relationship. Thus, this relationship was tested by fitting two models, an intercept only model and residuals as a function of ENSO. Of these two candidate models, the intercept only model was somewhat more parsimonious, separated from the ENSO model by only 2.95 AIC*_c_* units and receiving 81% of the support, suggesting there was little support for ENSO related variation in *δ* ^2^H_f_ in the data.

## Discussion

This was the first study to specifically examine the influence of species natural history on the relationship between *δ* ^2^H_p_ and *δ* ^2^H_f_ values at continental scales. Specifically, although there was a straightforward and overwhelming relationship between *δ* ^2^H_p_ and *δ* ^2^H_f_ values for North America, it was further identified that foraging guild and substrate were also key factors in this relationship. Recognition of additional ecological factors presented a significant step forward in the ability to use endogenous markers such as stable isotope ratios in feathers to assign geographical origins of passerines in North America and elsewhere.

The influence of foraging substrate on feather H isotopes, where it was observed that ground foragers generally had comparatively higher *δ* ^2^H, was not entirely surprising. Although the H isotopic differences among foragers appeared to be relatively small (10–36‰), compared to the global isotopic range in nature of over 250‰, these differences are significant enough to cause a substantive disparity in correctly assigning birds to previously established spatial isoscape models.

The precise cause of the H isotopic differences among migrant and resident foragers remains speculative, but has been previously observed. Hobson et al. [43] found systematic differences in *δ* ^2^H_f_ values between migrant ground-foraging Ovenbirds and canopy-foraging America Redstarts (*Setophaga ruticilla*) across several sites in the southern boreal of Canada. Similarly [44], found that ground-foraging species were enriched in deuterium, over shrub and canopy foragers in a tropical rainforest of Nicaragua. Ground foraging adult Wood Thrush (*Hylocichla mustelina*) in Georgia were more enriched in ^2^H than expected from the generalized algorithm used by [45].

The finding that migrant ground-foraging birds were more enriched in ^2^H than canopy foraging counterparts must be related to local diet or the diet during the period of feather growth or physiological differences between these foraging guilds. Several inter-related possibilities could account for these differences which ultimately result in enrichment in feather ^2^H. One possibility is that soil-level invertebrate prey may be comparatively enriched in ^2^H compared to shrub and canopy insects due to the evapotranspiration that can occur at the soil-atmosphere interface whereby isotopically light water is preferentially lost to the atmosphere compared to deeper water transmitted to leaves [46]. This would result, all things being equal, to feather ^2^H enrichment in ground foragers. The effect of evapotranspiration would be expected to be more pronounced in regions of high ambient temperature and/or low humidity. Alternatively, invertebrates used by ground-foraging birds (e.g. beetles, spiders) may simply represent a higher mean trophic level than herbivorous insects associated with the canopy and, if insect *δ* ^2^H correlates positively with trophic level, ground-foraging birds would be enriched in ^2^H over canopy foraging birds (e.g. [47]).

In contrast, resident ground foragers had lower *δ* ^2^H_f_ values than resident shrub or canopy foragers. This could be due to growth of feathers earlier in the season whereby ground-based diets are more influenced by winter or spring precipitation that is typically more depleted in ^2^H [48]. Migratory status is often linked to timing of molt and thus may in turn influence *δ* ^2^H_f_. Resident species in North America tend to breed earlier than migrants and subsequently molt earlier in the year. This may predispose residents to having lower *δ* ^2^H_f_ values if winter or spring precipitation made a greater contribution to foodweb *δ* ^2^H values (e.g. earlier plants and insects). Neotropical migrants in North America typically replace flight feathers before migration primarily during July and August. Clearly, possible causal mechanisms underlying the observed differences in *δ* ^2^H_f_ due to timing of molt and foraging substrate remain speculative and will require more research.

As several authors have indicated, differences in the algorithms linking *δ* ^2^H_p_ and *δ* ^2^H_f_ values for North America are not unexpected [1], [19], [25], [28], [30]. The challenge has been to refine the strong linkage between these factors. This study provides new evidence of the need for further investigation into how factors such as microhabitat characteristics and foraging guild drive variation in the relationship between *δ* ^2^H_f_ and *δ* ^2^H_p_. The results of this study are therefore encouraging and underline the fact that the more one can account for isotopic variability in feathers (and other tissues), the greater precision one might expect in assigning birds and other animals to geographic origin using appropriate empirical algorithms.

This study provides renewed and strong support for the enduring relationship between *δ* ^2^H_p_ and *δ* ^2^H_f_ values for North America and its application to geographic assignments to origin. Thus, challenges to the H isotope approach may be unnecessarily alarmist, particularly where North American passerines are concerned [26]. Several authors have argued that the variance associated with this precipitation-tissue H isotope relationship completely undermines the ability to accurately define origins of migratory birds. For example [24], claimed fundamental limits to the ability to assign migratory wildlife to origin are due to the temporal variation in precipitation isotope ratios as estimated from the Global Network of Isotopes in Precipitation database [24]. However, the clear lack of large systematic departures of the regression residuals from the long-term isoscape predictions (Figure 5), suggested that temporal variations in the GNIP data base may not be an appropriate measure of variance inherent in the assignment process. Indeed, high annual variation in precipitation ^2^H will be attenuated by averaging effects within the hydrologic cycle (lakes, streams, soil moisture, ground water), the foodweb, and the tendency for H isotopic variance to average out at higher trophic levels [26], [49]. Indeed, the results of applying the assignment algorithm to origins to the external validation data set suggested that samples can be assigned to origin at *pre-specified* levels of accuracy. Thus, in our opinion, the question instead should be how best to handle known sources of variance in both *δ* ^2^H_p_ and *δ* ^2^H_f_ when assigning individuals and populations to probability of origin surfaces [9], [19], [26], [41], [42]. Thus, the distribution of residuals from the robust relationship between *δ* ^2^H_f_ and *δ* ^2^H_p_, likely provided a better reflection of the true variance inherent in assignments to origin than variation *δ* ^2^H in precipitation represented in the GNIP isoscape data.

## Supporting Information

Table S1 List of species sampled for stable hydrogen isotope analysis of feathers and associated categorical factors used in models examining variation in *δ* ^2^H_f_.
(DOC)

Table S2 AIC_c_ based model selection statistics for the 25 a priori candidate models used to examine variation in δ2Hf. *K* = number of parameters, AIC_c_ = Akaike's Information Criterion corrected for small sample size, ΔAIC*c* = difference in AIC_c_ between model and highest ranked model, and ω_i_ = AIC weight.
(DOC)
