# Supplementary material for: Linking Hydrogen (δ 2H) Isotopes in Feathers and Precipitation: Sources of Variance and Consequences for Assignment to Isoscapes
Source: PLoS One. 2012 Apr 11;7(4):e35137. doi: 10.1371/journal.pone.0035137 (PMC3324428; doi:10.1371/journal.pone.0035137)
Supplement: Table S1 — List of species sampled for stable hydrogen isotope analysis of feathers and associated categorical factors used in models examining variation in δ 2Hf. (DOC) [file pone.0035137.s001.doc]

| AOU code | Common Name | Scientific Name | Migratory  Guild | Foraging Guild | Foraging Substrate | Habitat | n |
| --- | --- | --- | --- | --- | --- | --- | --- |
| ABTO | Abert's Towhee | *Melozone aberti* | Resident | Omnivore | Ground | Upland | 3 |
| AMRE | American Redstart | *Setophaga ruticilla* | Neotropical | Insectivore | Non-Ground | Upland | 18 |
| AMRO | American Robin | *Turdus migratorius* | Short distance | Omnivore | Non-Ground | Upland | 10 |
| AUWA | Yellow-rumped Warbler | *Setophaga coronata* | Short distance | Insectivore | Non-Ground | Upland | 6 |
| BAWW | Black-and-white Warbler | *Mniotilta varia* | Neotropical | Insectivore | Non-Ground | Upland | 1 |
| BCCH | Black-capped Chickadee | *Poecile atricapillus* | Resident | Insectivore | Non-Ground | Upland | 5 |
| BEWR | Bewick's Wren | *Thryomanes bewickii* | Short distance | Insectivore | Ground | Upland | 2 |
| BWWA | Blue-winged Warbler | *Vermivora pinus* | Neotropical | Insectivore | Non-Ground | Upland | 1 |
| CACH | Carolina Chickadee | *Poecile carlinensis* | Resident | Insectivore | Non-Ground | Upland | 5 |
| CARW | Carolina Wren | *Thryothorus ludovicianus* | Resident | Insectivore | Non-Ground | Upland | 9 |
| CHSP | Chipping Sparrow | *Spizella passerina* | Short distance | Omnivore | Ground | Upland | 9 |
| COYE | Common Yellowthroat | *Geothlypis trichas* | Neotropical | Insectivore | Non-Ground | Aquatic | 41 |
| CSWA | Chestnut-sided Warbler | *Setophaga pensylvanica* | Neotropical | Insectivore | Non-Ground | Upland | 2 |
| ETTI | Tufted Titmouse | *Baeolophus bicolor* | Resident | Omnivore | Non-Ground | Upland | 2 |
| GRCA | Gray Catbird | *Dumetella carolinensis* | Short distance | Omnivore | Ground | Upland | 2 |
| GWWA | Golden-winged Warbler | *Vermivora chrysoptera* | Neotropical | Insectivore | Non-Ground | Upland | 33 |
| HETH | Hermit Thrush | *Catharus guttatus* | Short distance | Omnivore | Ground | Upland | 3 |
| HOSP | House Sparrow | *Passer domesticus* | Resident | Omnivore | Ground | Upland | 1 |
| HOWA | Hooded Warbler | *Setophaga citrina* | Neotropical | Insectivore | Non-Ground | Upland | 4 |
| HOWR | House Wren | *Troglodytes aedon* | Short distance | Insectivore | Non-Ground | Upland | 1 |
| KEWA | Kentucky Warbler | *Geothlypis formosa* | Neotropical | Insectivore | Ground | Upland | 35 |
| MGWA | MacGillivray's Warbler | *Geothlypis tolmiei* | Neotropical | Insectivore | Non-Ground | Upland | 18 |
| NAWA | Nashville Warbler | *Oreothlypis ruficapilla* | Neotropical | Insectivore | Non-Ground | Upland | 1 |
| NOCA | Northern Cardinal | *Cardinalis cardinalis* | Resident | Omnivore | Ground | Upland | 13 |
| OCWA | Orange-crowned Warbler | *Oreothlypis celata* | Short distance | Insectivore | Non-Ground | Upland | 2 |
| ORJU | Dark-eyed Junco | *Junco hyemalis* | Short distance | Omnivore | Ground | Upland | 10 |
| OVEN | Ovenbird | *Seiurus aurocapilla* | Neotropical | Insectivore | Ground | Upland | 52 |
| RCSP | Rufous-crowned Sparrow | *Aimophila ruficeps* | Resident | Omnivore | Ground | Upland | 2 |
| REVI | Red-eyed Vireo | *Vireo olivaceus* | Neotropical | Insectivore | Non-Ground | Upland | 2 |
| RUBL | Rusty Blackbird | *Euphagus carolinus* | Short distance | Insectivore | Ground | Aquatic | 17 |
| SOSP | Song Sparrow | *Melospiza melodia* | Short distance | Omnivore | Non-Ground | Aquatic | 20 |
| SPPI | Sprague's Pipit | *Anthus spragueii* | Neotropical | Insectivore | Ground | Upland | 3 |
| SWTH | Swainson's Thrush | *Catharus ustulatus* | Neotropical | Omnivore | Ground | Upland | 77 |
| TRES | Tree Swallow | *Tachycineta bicolor* | Short distance | Insectivore | Non-Ground | Aquatic | 30 |
| VEER | Veery | *Catharus fuscescens* | Neotropical | Omnivore | Ground | Upland | 17 |
| WEVI | White-eyed Vireo | *Vireo griseus* | Neotropical | Insectivore | Non-Ground | Upland | 16 |
| WIWA | Wilson's Warbler | *Cardellina pusilla* | Neotropical | Insectivore | Non-Ground | Aquatic | 7 |
| WOTH | Wood Thrush | *Hylocichla mustelina* | Neotropical | Omnivore | Ground | Upland | 34 |
| YBCH | Yellow-breasted Chat | *Icteria virens* | Neotropical | Omnivore | Non-Ground | Upland | 9 |
| YWAR | Yellow Warbler | *Setophaga petechia* | Neotropical | Insectivore | Non-Ground | Aquatic | 21 |
