# Supplementary material for: Linking Hydrogen (δ 2H) Isotopes in Feathers and Precipitation: Sources of Variance and Consequences for Assignment to Isoscapes
Source: PLoS One. 2012 Apr 11;7(4):e35137. doi: 10.1371/journal.pone.0035137 (PMC3324428; doi:10.1371/journal.pone.0035137)
Supplement: Table S2 — AICc based model selection statistics for the 25 a priori candidate models used to examine variation in δ2Hf. K = number of parameters, AICc = Akaike's Information Criterion corrected for small sample size, ΔAICc = difference in AICc between model and highest ranked model, and ωi = AIC weight. (DOC) [file pone.0035137.s002.doc]

| Model | *K* | AIC*_c_* | ΔAIC*_c_* | ω_i_ |
| --- | --- | --- | --- | --- |
| Int^a^ + *δ*^2^H_p_ + MG^b^ + FS^c^ + MG*FS | 8 | 4324.52 | 0 | 0.99 |
| Int + *δ*^2^H_p_ + MG + FS + MG* *δ*^2^H_p_ + FS* *δ*^2^H_p_ | 9 | 4334.74 | 10.22 | 0.01 |
| Int + *δ*^2^H_p_ + MG + FS | 6 | 4362.2 | 37.68 | 0 |
| Int + *δ*^2^H_p_ + MG + FS + FG + FG* *δ*^2^H_p_ | 8 | 4363.98 | 39.46 | 0 |
| Int + *δ*^2^H_p_ + FS + FG + AU^e^ | 6 | 4374.08 | 49.56 | 0 |
| Int + *δ*^2^H_p_ + FS + AU + FS*AU | 6 | 4378.51 | 53.98 | 0 |
| Int + *δ*^2^H_p_ + FS + AU | 5 | 4380.6 | 56.08 | 0 |
| Int + *δ*^2^H_p_ + MG + FG + MG*FG | 8 | 4381.2 | 56.68 | 0 |
| Int + *δ*^2^H_p_ + Age^d^ + FS | 6 | 4385.47 | 60.95 | 0 |
| Int + *δ*^2^H_p_ + FS + FG | 5 | 4386.02 | 61.5 | 0 |
| Int + *δ*^2^H_p_ + MG + AU + MG*AU | 7 | 4391.12 | 66.6 | 0 |
| Int + *δ*^2^H_p_ + MG + AU | 6 | 4391.38 | 66.85 | 0 |
| Int + *δ*^2^H_p_ + FS | 4 | 4391.51 | 66.99 | 0 |
| Int + *δ*^2^H_p_ + FG + AU + FG*AU | 6 | 4394.01 | 69.48 | 0 |
| Int + *δ*^2^H_p_ + AU | 4 | 4406.29 | 81.77 | 0 |
| Int + *δ*^2^H_p_ + MG + FG | 6 | 4406.86 | 82.34 | 0 |
| Int + *δ*^2^H_p_ + AU + FG | 5 | 4408.15 | 83.63 | 0 |
| Int + *δ*^2^H_p_ + Age + MG | 7 | 4408.83 | 84.31 | 0 |
| Int + *δ*^2^H_p_ + MG | 5 | 4410.37 | 85.85 | 0 |
| Int + *δ*^2^H_p_ + Age + *δ*^2^H_p_*Age | 7 | 4418.73 | 94.21 | 0 |
| Int + *δ*^2^H_p_ + Age | 5 | 4442.6 | 118.07 | 0 |
| Int + *δ*^2^H_p_ + Age + FG | 6 | 4442.64 | 118.12 | 0 |
| Int + *δ*^2^H_p_ | 3 | 4447.14 | 122.62 | 0 |
| Int + *δ*^2^H_p_ + FG | 4 | 4447.69 | 123.17 | 0 |
| Int | 2 | 5273.35 | 948.83 | 0 |

^a^ Int = Intercept

^b^ MG = Migratory Guild

^c^ FS = Foraging Substrate

^d^ Age = Age class

^e^ AU = Aquatic versus Upland
